# Toward a cure – Advancing HIV/AIDs treatment modalities beyond antiretroviral therapy: A Review

**DOI:** 10.1097/MD.0000000000038768

**Published:** 2024-07-05

**Authors:** Esther Ugo Alum, Daniel Ejim Uti, Okechukwu Paul-Chima Ugwu, Benedict Nnachi Alum

**Affiliations:** a Department of Research and Publications, Kampala International University, Kampala, Uganda.

**Keywords:** adherence, antiretroviral therapy, drug resistance, HIV/AIDS, immune system, immunotherapy, latency-reversing agents

## Abstract

Antiretroviral therapy, also known as antiretroviral therapy (ART), has been at the forefront of the ongoing battle against human immunodeficiency virus/acquired immunodeficiency syndrome (HIV/AIDs). ART is effective, but it has drawbacks such as side effects, medication resistance, and difficulty getting access to treatment, which highlights the urgent need for novel treatment approaches. This review explores the complex field of HIV/AIDS treatment, covering both established alternative treatment modalities and orthodox antiretroviral therapy. Numerous reliable databases were reviewed, including PubMed, Web of Science, Scopus, and Google Scholar. The results of a thorough literature search revealed numerous therapeutic options, including stem cell transplantation, immunotherapy, gene therapy, latency reversal agents, and pharmaceutical vaccinations. While gene therapy has promise for altering cellular resistance to infection and targeting HIV-positive cells, immunotherapy treatments seek to strengthen the immune system’s ability to combat HIV. Latency reversal agents offer a promising method of breaking the viral latency and making infected cells vulnerable to immune system destruction or antiretroviral drugs. Furthermore, there is potential for improving immune responses against HIV using medical vaccinations. This review stresses the vital significance of ongoing research and innovation in the hunt for a successful HIV/AIDS treatment through a thorough examination of recent developments and lingering challenges. The assessment notes that even though there has been tremendous progress in treating the illness, there is still more work to be done in addressing current barriers and investigating various treatment options in order to achieve the ultimate objective of putting an end to the HIV/AIDS pandemic.

## 1. Introduction

The HIV/AIDs continue to be major threats to public health around the world. HIV/AIDS is still a serious threat despite tremendous advancements in our knowledge of the virus and the creation of efficient treatment plans. This is especially true in low-income and middle-income countries where access to medical care may be restricted.^[1,2]^ Antiretroviral therapy (ART), which has transformed patient care and dramatically improved outcomes, is essential to the management of HIV/AIDS. Though ART has changed HIV status from a death sentence to a manageable chronic disease, it is not a cure. ART is not without its drawbacks, though, including as the rise in drug-resistant strains, adverse effects, and obstacles to access in environments with limited resources.^[3,4]^ Similarly, viral reservoirs, drug resistance, and lifelong adherence to medication are mentioned as some of the reasons alternative treatment strategies are necessary without delay.^[5]^ Given these difficulties, research into new treatment modalities that may provide long-term viral control, less dependence on lifetime medicine, and eventually an HIV cure is necessary. The aim of this review is to investigate the present frontiers of HIV/AIDS research and reveal promising directions that hold the promise of advancing to a cure. A thorough search of the literature was done, with particular attention to reliable databases such as PubMed, Google Scholar, Scopus, Web of Science, and others. The findings indicate that stem cell transplantation, gene therapy, immunotherapy, latency reversal agents (LRAs), and pharmaceutical vaccines are promising alternative therapeutic approaches.^[6]^ This article attempts to provide light on the changing landscape of HIV/AIDs management by synthesizing current research findings and addressing the complications inherent in HIV/AIDS treatment. By gaining a thorough understanding of current obstacles and prospects, we can steer clear of them and take a more efficient and long-lasting approach to tackling this persistent worldwide health emergency.

## 2. Review methodology/search strategy

The study did an exhaustive search using various electronic databases such as PubMed/MEDLINE, Embase, Web of Science, Scopus, Google Scholar, and Cochrane Library to identify other ways to treat HIV/AIDS other than antiretroviral therapy. The study included articles published in English-language peer-reviewed journals, excluding those that solely focused on antiretroviral therapy without considering other medications or cures. We screened the titles and abstracts of the identified articles, and collected data using a standardized form. We used articles published between 2015 and 2024. The analysis compiled and collated new developments in HIV/AIDS treatments beyond antiretroviral therapy into a comprehensive report. In the context of treatment modalities, we grouped them by themes, like viral reservoir clearance, immunotherapy, gene therapy, and combination therapy. We have outlined all the results concerning efficacy, safety, and any possible complications associated with each treatment modality. We highlighted the gaps and potential research areas.

## 3. Overview of HIV/AIDS

The virus known as HIV affects the immune system, primarily focusing on CD4 cells, which are essential for warding off infections. The advanced stage of HIV infection known as acquired immunodeficiency syndrome (AIDS) results in significant immune system damage and increased vulnerability to malignancies and opportunistic infections.^[7]^ HIV is mainly passed from mother to child during childbirth or breastfeeding, through sharing contaminated needles, and through unprotected sexual contact.^[8]^ Additionally, infusions of infected blood can spread it, though these days, screening procedures have made this uncommon.^[9]^ The signs and symptoms of HIV differ according on the infection stage. People may have flu-like symptoms in the early stages, including fever, lethargy, and enlarged lymph nodes.^[10]^ HIV, however, can go years without showing any symptoms. If therapy is not received, HIV develops into AIDS, which is characterized by a severe immunological deficit and the emergence of malignancies or opportunistic infections.^[11]^

The main treatment for HIV is called antiretroviral therapy (ART), which consists of a cocktail of drugs that prevent the virus from replicating and help the immune system heal and operate normally. ART can effectively control HIV, allowing infected patients to live long and healthy lives, even though therapy cannot cure the virus.^[12]^ Promoting safe sexual behaviors, supporting routine HIV testing, giving injecting drug users access to sterile needles, and reducing mother-to-child transmission through interventions during pregnancy, childbirth, and lactation are some examples of prevention efforts.^[13]^ HIV/AIDS management and prevention have made great strides, but the disease still poses a threat to global public health, especially in low-income and middle-income nations where access to services for prevention and treatment may be restricted.^[14]^ Sustained endeavors are imperative to curtail novel infections, enhance care accessibility, and finally accomplish the objective of terminating the HIV/AIDS pandemic.

## 4. Antiretroviral therapy (ART): restrictions and difficulties

Millions of people worldwide have seen a major improvement in their quality of life and lifespan courtesy of antiretroviral therapy (ART), which has completely changed how HIV/AIDS is managed. ART is effective, but it also has a number of drawbacks and difficulties. The aforementioned constraints and difficulties highlight the necessity of continuous investigation, creativity, and all-encompassing medical methodologies in order to maximize therapeutic results and elevate the standard of living for HIV/AIDS patients. The challenges faced by HIV/AIDS patients include drug resistance, side effects, treatment adherence, access and cost, long-term complications, viral reservoirs, and stigma. Drug-resistant strains can evolve due to fast mutations, while side effects like fatigue, nausea, and metabolic problems can impact health and treatment compliance.^[15]^ Access to ART is difficult, especially in low-resource environments. Long-term complications like osteoporosis, renal impairment, and cardiovascular disorders require comprehensive healthcare practices. Eliminating viral reservoirs and addressing stigma are crucial for achieving a cure.^[16]^ Addressing these challenges is essential for promoting HIV testing, treatment, and long-term ART adherence.

## 5. Inherent need for alternative HIV treatment approaches

Alternative curative methods are desperately needed, even though antiretroviral medication (ART) has greatly improved the prognosis and quality of life for those with HIV. Effectively stopping HIV replication, antiretroviral therapy (ART) carries a lifelong risk of medication resistance, side effects, and long-term health issues.^[15]^ Furthermore, because of infrastructure, stigma, and cost, access to ART is still restricted in many regions of the world. Instead of requiring lifelong antiretroviral therapy, alternative HIV cure options seek to achieve prolonged viral remission or elimination.^[17]^ Vaccines intended to enhance the immune system’s defense against HIV, either by preventing infection or by limiting the virus’s ability to replicate in those who have already contracted it.^[18]^ Gene therapy involves targeting HIV reservoirs or immune cells with gene-editing tools such as CRISPR-Cas9 in order to alter them and maybe make them resistant to viral replication or make it possible for the immune system to identify and destroy infected cells.^[19]^ Immunotherapy are treatments that boost the body’s defenses against HIV, include immune checkpoint inhibitors and adoptive cell transfer, in which patients receive infusions of immune cells that have been genetically modified to recognize and eradicate HIV-infected cells.^[20]^ Furthermore, Latency-Reversing Agents (LRAs) are substances that try to revive dormant HIV reservoirs, exposing infected cells to immune-mediated clearance or rendering them apparent to the immune system.^[21]^ Combination therapies involve combining many strategies, such as gene therapy and immunotherapy, to target various components of the HIV lifecycle and reservoirs at the same time. Immune-based therapies involve HIV neutralization or improved immune responses against the virus by the use of antibodies or other immunological-based medicines.^[22]^

## 6. Methods of immunotherapy for HIV management

Although ART has demonstrated efficacy in limiting viral replication in cells, its administration has been hampered by toxicity, resistance, and inadequate penetration into immune privileged areas, such as the central nervous system (CNS). Particularly, the neurotoxic effects, inconsistent CNS penetration, and viral escape in the CNS have been the main drawbacks of ART in the CNS.^[23]^ Promising as an area in HIV care, immunotherapy works to strengthen and modify the body’s defenses against the HIV. In contrast to the primary goal of traditional ART, immunotherapy aims to fortify the immune system’s ability to combat HIV.^[24]^ Some of the immunotherapy strategies under investigation for HIV management are here in highlighted. Vaccinations with therapeutic properties work to alert the immune system to identify and combat HIV-infected cells. These vaccines frequently use DNA or viral proteins to elicit an immune response.^[25]^ Additional research is required to maximize the effectiveness of early treatment studies, even if they have showed some promise in lowering viral load and slowing the development of the disease.

Naturally occurring antibodies known as “broadly neutralizing antibodies” (bNAbs) have the ability to neutralize a variety of HIV strains. They may be used to treat HIV infections as well as prevent them. To provide transient protection against HIV, monoclonal antibodies made from bNAbs are being produced for passive immunization.^[26]^ Furthermore, vaccinations are being used to induce the synthesis of bNAbs. Checkpoint Inhibitors act by obstructing inhibitory pathways that stifle immunological responses, checkpoint inhibitors aim to maximize the potential of the immune system. Checkpoint inhibitors are designed to help HIV-positive people regain their immune function by going after molecules like PD-1 or CTLA-4. There are current clinical trials examining the security and effectiveness of checkpoint inhibitors in HIV.^[27]^

### 6.1. Gene therapy

Gene therapy entails altering a patient’s immune cells to improve their capacity to identify and eradicate HIV-positive cells. It is being investigated how to modify immune cells’ genetic codes to make them resistant to HIV infection using methods like CRISPR/Cas9. Gene therapy may also entail the transfer of immune cells that have been modified to recognize and eliminate HIV-infected cells. Compounds known as immune modulators control the immunological response by either increasing immune activation or decreasing inflammation.^[28]^ The potential of agents like interleukins, interferons, and toll-like receptor agonists to increase antiviral immunity in HIV-positive people is being studied. According to Clutton et al,^[29]^ interleukin-10 enhances the production of CD8^+^T cells. These strategies seek to strengthen the immune system’s capacity to curb HIV replication and stop the spread of the illness.

With its novel approaches to combating the virus, gene therapy has the potential to completely transform the way that HIV is managed. Gene-editing immune cells, including T cells, to express receptors that identify and combat HIV-infected cells more successfully is a major gene therapy approach. Furthermore, HIV DNA within infected cells may be directly targeted by gene-editing methods like CRISPR-Cas9, which could disable or prevent the virus from replicating. Gene therapy is a promising new direction in the continuous search for efficient HIV treatment, even though there are still obstacles to be overcome, such as safety issues and the requirement for more study. Gene therapy may eventually provide a workable cure for the virus.^[28]^

#### 6.1.1. Cas9/CRISPR technology

The application of CRISPR/Cas9 technology to HIV control shows enormous promise. Since the HIV integrates into the host’s DNA, it is difficult to destroy with conventional antiretroviral therapy (ART) alone. A unique way to combat HIV is provided by the genome-editing tool CRISPR/Cas9, which targets and modifies the viral genome within infected cells.^[30]^ The following are some applications of CRISPR/Cas9 technology in HIV management:

1. *Editing the HIV genome:* CRISPR/Cas9 may be designed to specifically target sequences in the HIV genome, causing disruptions to vital genes necessary for the virus to replicate. CRISPR/Cas9 can make a virus nonfunctional by inducing mutations or deletions in crucial viral DNA sequences, which stops the virus from replicating and infecting new cells.^[31]^
2. *Latent HIV eradication:* The persistence of latent reservoirs of the virus, which lie dormant within infected cells and are immune system and antiretroviral therapy resistant, is one of the greatest obstacles to HIV treatment. By triggering viral gene expression or causing cell death in latently infected cells, CRISPR/Cas9 has the ability to specifically target and eradicate these latent HIV reservoirs.^[32]^
3. *Host cell modification:* In order to give resistance to HIV infection, host cells can also be modified using CRISPR/Cas9. For instance, cells can be rendered resistant to HIV infection by modifying the CCR5 gene, which codes for a co-receptor that HIV uses to enter immune cells. Clinical trials have exhibited this strategy, wherein HIV patients were given infusions of their own immune cells that had been altered through CRISPR/Cas9 to cause disruption of the CCR5 gene.^[33]^
4. *Viral load reduction:* CRISPR/Cas9 has the ability to effectively target and disrupt viral genes in HIV-positive patients, thereby reducing the viral load and subsequently the risk of disease progression and transmission.^[33]^
5. *Combination medications:* CRISPR/Cas9 technology can be used to create synergistic approaches for HIV care by combining it with currently available antiretroviral medications as well as other cutting-edge treatments like gene therapy and immunotherapy. These combination medications have the potential to surpass the limitations of existing HIV therapies and improve treatment efficacy.^[34]^

#### 6.1.2. HIV management using zinc finger nucleases (ZFNs) and transcription activator-like effector nucleases (TALENs)

HIV management is one of the many uses for the genome-editing tools ZFNs and TALENs that have been investigated. Since HIV is a retrovirus, it is difficult to fully eliminate since it incorporates its genetic material into the host DNA. On the other hand, novel approaches of altering host cells to provide resistance to HIV infection or upsetting the viral genome are presented by genome-editing technologies such as ZFNs and TALENs.^[35]^ It’s important to remember that although preclinical research and early-phase clinical trials have demonstrated the potential of ZFNs and TALENs, their therapeutic value in HIV therapy is currently being investigated. Before HIV medication is widely used, issues like delivery strategies, scalability, and off-target effects must be resolved. How they can be applied to HIV management is as follows:

##### i. HIV gene disruption:

Certain sequences within the HIV genome can be targeted and cleaved by ZFNs and TALENs. These nucleases can stop viral replication by creating double-stranded breaks in important viral genomic regions. Targeting crucial genes such as pol, env, or gag, for example, can hinder the creation of viable viral particles.^[36]^

##### ii. Host cell modification:

Changing host cells to give them resistance to HIV infection is another strategy. Genes encoding co-receptors or other cellular components necessary for HIV entry and replication can be disrupted in order to accomplish this. For instance, co-receptor CCR5 is necessary for HIV entrance into CD4+ T cells. ZFNs or TALENs can disrupt the CCR5 gene, making cells resistant to specific HIV strains.^[37]^

##### iii. Creation of HIV-resistant cells:

Hematopoietic stem cells (HSCs) or immune cells such as T cells can be modified to give HIV resistance using ZFNs and TALENs. The patient may subsequently have a stem cell transplant or adoptive cell therapy to reintroduce these altered cells. This strategy, known as gene therapy for HIV, has been investigated in clinical trials with encouraging outcomes.^[38]^

##### iv. Targeted gene activation or repression:

ZFNs and TALENs can be used to control gene expression in addition to direct genome-editing. It may be possible to stop the transmission of the virus or improve antiviral immunity by altering the expression of host components involved in HIV replication or the immune response.^[39]^

#### 6.1.3. HIV management using lentiviral vector-mediated gene therapy

Lentiviral vector-mediated gene therapy has the potential to control HIV by inserting protective genes into vectors, targeting immune cells like T cells or HSCs, boosting immune responses, and disrupting HIV co-receptors like CCR5.^[40]^ However, challenges include the need for secure delivery techniques, concerns over immunological reactions, off-target effects, and the possibility of mutagenesis. Additionally, the development of resistance to gene-based therapies and the long-term stability of therapeutic benefits remain significant factors. Clinical trials are ongoing to assess the safety and effectiveness of this therapy, evaluating approaches like T cell, HSC, and immune cell gene alteration.^[41]^ Future research could focus on refining vector design, enhancing delivery techniques, and exploring combination strategies with additional antiviral treatments or immunomodulatory drugs.

### 6.2. LRAs, or latency reversal agents, in HIV treatment

One exciting new development in the ongoing fight against HIV/AIDs is the use of latency reversal agents, or LRAs. The virus that causes AIDs, HIV, has the unusual capacity to lurk inside immune cells in the body, avoiding identification and therapy. The covert activity referred to as latency poses a significant challenge to finding an effective treatment for HIV infection. Nevertheless, the idea of latency reversal presents a possible way to overcome this difficulty.

Essentially, latency reversal entails rousing the quiescent HIV-positive cells, evicting them from their hiding locations, and rendering them vulnerable to the immune system’s assault or antiretroviral medication.^[42]^ Compounds or medications known as LRAs are made to start this process of awakening, which effectively flushes the virus out of its hiding places and makes it susceptible to removal.^[43]^ The knowledge that although ART now available efficiently reduces HIV replication, it is not a cure for the infection, which is the basis for the argument behind the use of LRAs. Resting CD4^+^ T cells are long-lived immune cells that have the ability to harbor HIV in a latent state. A functional cure for HIV/AIDs is significantly hampered by these latent reservoirs, which act as a continuous source of viral rebound in the event that treatment is stopped. Through their ability to target and deplete these latent reservoirs, LRAs present a promising addition to current therapeutic approaches. LRAs reveal latent HIV-infected cells to immune system monitoring mechanisms and enable antiretroviral medications to eradicate the virus. Finding LRAs that can specifically reactivate latent HIV without overstimulating the immune system or causing toxicity is the difficult part, though.^[32]^ According to Wang et al^[44]^ various types of drugs, such as protein kinase C agonists, histone deacetylase inhibitors (HDACis), Toll-like receptor agonists, and bromodomain and extraterminal domain inhibitors (BETis), have been studied as possible LRAs. Different strategies are employed by each family of LRAs to cause disruptions in the molecular pathways that sustain HIV latency. It has proven difficult to convert the promise of LRAs into clinical success, despite significant advancements in preclinical research. Pitman et al^[45]^ cited obstacles in the way of LRA clinical trials to include low efficacy, off-target effects, and the failure to achieve long-term viral suppression in the absence of concurrent antiretroviral therapy. Concerns about viral reservoirs reseeding and the possibility of viral comeback after stopping LRA treatment are other important factors to take into account. Ait-Ammar et al^[46]^ opined that the creation of LRAs for HIV management necessitates a comprehensive strategy that tackles important clinical, scientific, and practical issues. This entails improving treatment regimens, finding safer and more effective LRAs, enhancing our knowledge of HIV latency and reservoir dynamics, and investigating combination strategies that work in concert with already available antiretroviral medications. LRAs have great potential as a key element of HIV treatment plans in the future, despite the difficulties associated with them. Even if a permanent cure for HIV/AIDS is still a long way off, the scientific community’s dedication to finding novel solutions, such as latency reversal, is evident in their efforts to address one of the biggest public health issues of our day.

## 7. Medicinal vaccines for HIV treatment

A promising field of study aiming at managing HIV infection and lowering the need for continuous ART is therapeutic vaccinations. The goal of these vaccinations is to help contain the virus and stop the spread of the disease by encouraging the immune system to identify and target HIV-infected cells.^[47]^ Therapeutic HIV vaccines aim to modify the immune response in infected individuals, enhancing their ability to regulate the virus, lower viral load, and delay infection spread. These vaccines can target T cells, which activate immune cells, and antibodies, which stimulate the generation of broadly neutralizing antibodies. Some vaccines combine T cell and antibody-based techniques.^[48]^ Clinical trials assess the immunogenicity, safety, and effectiveness of these vaccines, but sustained viral suppression without antiretroviral therapy remains a challenge.

Therapeutic HIV vaccines face challenges due to the genetic diversity of HIV, its immune-evading strategies, and optimal design. Despite these obstacles, research continues to improve vaccine design, find new targets, and boost immune responses. Advancements in delivery methods, such as nanoparticles and viral vectors, could potentially improve vaccination efficacy.^[47]^

## 8. Stem cell donation for HIV treatment

Because of Timothy Ray Brown, otherwise known as the “Berlin Patient,” who became the first person to be cured of HIV after receiving a stem cell transplant from a donor who had a rare genetic mutation known as CCR5 delta 32, which makes cells resistant to HIV infection, stem cell transplantation (SCT) has gained attention in HIV management.^[49]^ Stem cell transplantation is not widely accepted for treating HIV due to its high risk, complexity, and availability of compatible donors. It is quite uncommon to find a compatible donor that carries the CCR5 delta 32 mutation, like Timothy Ray Brown did. Only a very tiny portion of the population carries this mutation. It is also expensive, making it difficult for many HIV-positive individuals to afford. More so, stem cell transplantation raises ethical questions, especially when using risky experimental techniques when safer alternatives are available.^[50]^ Notwithstanding these obstacles, stem cell transplantation is still being investigated as a possible HIV treatment approach. Alternative stem cell sources, such as induced pluripotent stem cells (iPSCs), and gene-editing methods to give transplanted cells HIV resistance are being studied in certain ongoing trials.

## 9. Conclusion

HIV/AIDs therapy must be approached holistically, incorporating both conventional and nonmethods of care. ART has played a significant role in reducing viral replication and enhancing patient outcomes; yet, its drawbacks highlight the necessity for ongoing investigation and testing of substitute approaches. The development of stem cell transplantation, immunotherapy, gene therapy, LRAs, and pharmaceutical vaccines presents novel approaches to attaining long-term viral control and possibly even an HIV/AIDs cure. With these encouraging methods, the problems of medication resistance, side effects, and barriers to access related to ART may be resolved. But it is important to recognize the challenges and complications that come with creating and putting these innovative treatments into practice. It is necessary to handle issues such ensuring safety, efficacy, and affordability as well as ethical considerations and inequality in access to care. In order to fully use these new therapy methods, continued investment in research, innovation, and cooperation will be necessary. Together with a dedication to tackling institutional, social, and economic obstacles, scientific and technological advancements can help us move closer to our common objective of putting an end to the HIV/AIDs pandemic.

## Acknowledgments

We are grateful to Kampala International University for its support.

## Author contributions

**Conceptualization:** Esther Ugo Alum.

**Data curation:** Esther Ugo Alum.

**Methodology:** Esther Ugo Alum, Daniel Ejim Uti, Okechukwu Paul-Chima Ugwu.

**Validation:** Esther Ugo Alum, Benedict Alum.

**Writing – original draft:** Esther Ugo Alum, Daniel Ejim Uti, Okechukwu Paul-Chima Ugwu, Benedict Alum.

**Writing – review & editing:** Esther Ugo Alum, Daniel Ejim Uti, Okechukwu Paul-Chima Ugwu, Benedict Alum.

**Investigation:** Daniel Ejim Uti.

**Visualization:** Daniel Ejim Uti, Benedict Alum.

**Resources:** Okechukwu Paul-Chima Ugwu, Benedict Alum.

**Supervision:** Okechukwu Paul-Chima Ugwu.
